# 2019: An Awesome Year for *Pharmaceuticals*

**DOI:** 10.3390/ph13010006

**Published:** 2020-01-01

**Authors:** Jean Jacques Vanden Eynde

**Affiliations:** Editor-in-Chief, MDPI AG, St. Alban-Anlage 66, 4052 Basel, Switzerland; jean-jacques.vandeneynde@ex.umons.ac.be; Tel.: +32-2-355-81-61

Ending the year is an opportunity to reflect on the past, in particular the past twelve months. Without any exaggeration, we may say that our Journal *Pharmaceuticals* is growing [1] in a wonderful and successful way. Since 2015 the number of papers published annually jumped from 45 to 186 in 2019. That impressive number constitutes a tied record with Volume 3 edited in 2010. As a result, starting in 2020, *Pharmaceuticals* will be published on a monthly basis. Another milestone has been achieved by releasing the 1000th article in September, a contribution of B. O’Brien, S.J. Mathew, *et al.* [2]. Mention must also be made to the 17 Special Issues of this past year, three of them are the subject of printed books. The development of our Journal is, indubitably, the result of the dynamic and enthusiastic work of the editorial staff and all the editorial board members. Let me also underline that in 2019 five eminent female scientists, namely Martina Benesova (Heidelberg, Germany), Micheline Draye (Le Bourget du Lac, France), María Jesús Pérez Pérez (Madrid, Spain), Amelia Pilar Rauter (Lisboa, Portugal), and Svetiana Tsogoeva (Erlangen, Germany), joined the board [3]. 

In order to establish its worldwide reputation, our Journal acted in a media partnership and as a sponsor for numerous colloquia in different countries. Among them, we had the opportunity to promote *Pharmaceuticals* during the 257th national meeting and exhibition of the American Chemical Society in Orlando (FL, USA) in April. In August, we had the pleasure to announce that the 2019 *Pharmaceuticals* Travel Award [4] was granted to Jorge Henrique Ferreira Grilo, a Ph.D. student. Several other travel grants were also offered to reward young researchers and renowned scientists for their outstanding works. Such was the case during the 5th International Electronic Conference on Medicinal Chemistry [5], an event organized and sponsored by our Journal. That edition marked another noticeable milestone for *Pharmaceuticals*; indeed, it gathered a record of more than 400 authors and thousands of visitors. The 116 accepted abstracts were divided into three sessions: posters, presentations (soundless), and keynote presentations (videos with sound). It is noteworthy to address that conference speakers were evenly distributed between women and men. For each session, the scientific committee had the hard task of selecting the best work. Finally, travel grants were awarded to Elena González-Burgos, *et al.* for best poster [6]; Thines Kanagasundaram, *et al.* for best presentation [7]; and Edeildo Ferreira da Silva-Júnior, *et al.* for best keynote presentation [8]. The 6th edition of the series will be held on the web in November 2020. We are awaiting your submissions!

Do you remember my editorial [9] published in July 2017? In that note, I expressed my amazement because *Pharmaceuticals* had an excellent CiteScore™ (4.90 for 2016), as calculated by Elsevier, but was still awaiting its first journal impact factor, as estimated by Clarivate Analytics. Exceptional news was received in November—*Pharmaceuticals* has been accepted by World of Science™ (WoS™; Clarivate Analytics) to be included in the Science Citation Index Expanded (SCIE). This means that the first impact factor of our Journal will be announced in June 2020. 

To close this editorial, I would like to thank all our authors, reviewers, readers, and members of the editorial board and editorial offices for their confidence and continuous support. *Pharmaceuticals* could not be what it has become without your efforts and dynamism. May 2020 bring you 366 days of health, joy, and happiness.
